# Synergistic effects of earthworms and cow manure under reduced chemical fertilization modified microbial community structure to mitigate continuous cropping effects on Chinese flowering cabbage

**DOI:** 10.3389/fmicb.2023.1285464

**Published:** 2023-10-26

**Authors:** Fucheng Gao, Lin Ye, Xiaoguo Mu, Lei Xu, Zhenfang Shi, Yuan Luo

**Affiliations:** College of Wine and Horticulture, Ningxia University, Yinchuan, China

**Keywords:** biodiversity, Chinese flowering cabbage, earthworm, environmental factors, fertilizer, yield

## Abstract

The substitution of chemical fertilizers with organic fertilizers is a viable strategy to enhance crop yield and soil quality. In this study, the aim was to investigate the changes in soil microorganisms, soil chemical properties, and growth of Chinese flowering cabbage under different fertilization treatments involving earthworms and cow manure. Compared with the control (100% chemical fertilizer), CE (30% reduction in chemical fertilizer + earthworms) and CFE (30% reduction in chemical fertilizer + cow dung + earthworms) treatments at soil pH 8.14 and 8.07, respectively, and CFC (30% reduction in chemical fertilizer + cow manure) and CFE treatments increased soil organic matter (SOM), total nitrogen (TN), available nitrogen (AN), and available potassium (AK) contents. Earthworms and cow manure promoted the abundance of *Bacillus* and reduced that of the pathogens *Plectosphaerella* and *Gibberella*. The mantle test revealed that pH was not correlated with the microbial community. Random forest analysis verified that AN, SOM, and TN were important factors that jointly influenced bacterial and fungal diversity. Overall, the synergistic effect of earthworms and cow manure increased soil fertility and microbial diversity, thereby promoting the growth and development of Chinese flowering cabbage. This study enhanced the understanding of how bioregulation affects the growth and soil quality of Chinese flowering cabbage, and thus provided a guidance for the optimization of fertilization strategies to maximize the yield and quality of Chinese flowering cabbage while reducing environmental risks.

## 1. Introduction

Increasing application of fertilizers has resulted in adverse consequences on soil and water quality, biodiversity, climate, and human health. These effects are more pronounced than the corresponding increases in crop yields (Kour et al., 2020). In many intensive farming regions, the farmers struggle to accurately determine fertilizer requirements for crops and prioritize high yields. This often results in excessive use of fertilizers that exceeds the actual needs of the crops (Zhu et al., 2019). This problem is primarily attributed to inadequate fertilizer management, leading to reduced fertilizer efficiency and environmental contamination. Agricultural systems face the formidable challenge of meeting the mounting food demand while minimizing the harmful environmental impacts associated with intensive use of fertilizers, particularly nitrogen (N) fertilizers (Steffen et al., 2015). Excessive N fertilizer application adversely affects soil physicochemical properties and disrupts microbial communities, causing imbalance in the soil ecosystem (Wu et al., 2020). Overuse of fertilizers reduces soil microbial biomass by reducing microbial abundance and diversity. Additionally, excessive fertilization weakens the stability of microbial community and diminishes the interactions among microbial taxa (Zhang et al., 2013). These findings highlight the negative impacts of excessive fertilization on sustainable soil productivity and microbial ecology; for example, long-term excessive application of synthetic N fertilizers is associated with the degradation of soil microbial communities. In a study, long-term application of inorganic fertilizers augmented total soil organic carbon levels and significantly increased maize yields (Belay et al., 2002). However, the excessive use of chemical fertilizers leads to nitrate accumulation in vegetable products, thereby compromising food safety. In recent years, organic fertilizers have garnered considerable attention as a sustainable alternative to mitigate N losses and restore soil fertility. Combining organic fertilizers with low dose of chemical fertilizer can help in alleviating the adverse effects of overuse of chemical fertilizers. Numerous studies have demonstrated that the partial substitution of chemical fertilizers with organic fertilizers is a promising strategy for enhancing soil fertility and crop yield. For example, replacement of chemical fertilizers with organic counterparts augments the effectiveness of soil N and phosphorus, ultimately increasing cabbage yield (Xiao et al., 2022). Application of compost along with organic fertilizers can enhance soil fertility, increase fungal abundance and diversity, and promote the abundance of favorable microbial taxa (Kamaa et al., 2011; Jiao et al., 2021).

Decline in the yield and quality of Chinese flowering cabbage, resulting from frequent succession and uncontrolled fertilizer application, can be attributed to reduced soil porosity and water content and microbial community imbalance caused by excessive fertilization. Earthworms, as crucial organisms in soil ecosystem, play a pivotal role in soil structure formation, nutrient cycling, and organic matter decomposition (Siebert et al., 2019). Their activity enhances soil porosity, water infiltration, and nutrient availability. Furthermore, earthworms facilitate the transportation and blending of organic material in soil, promoting the interaction between organic fertilizers and soil microbial communities (Wang et al., 2021). Studies have demonstrated that the activity of earthworms alters the abundance and proportions of bacteria, fungi, and actinomycetes, thereby altering the microbial community structure (Medina-Sauza et al., 2019). In addition, earthworm activity can influence soil pH and the availability of trace elements, thereby improving microbial activity and community structure. This, in turn, increases the abundance of beneficial soil microorganisms, enhances bacterial community diversity, and promotes microbially mediated organic matter cycling (Cai et al., 2020). Soil microbial communities serve as essential drivers of nutrient cycling and soil fertility and play a fundamental role in organic matter decomposition, N fixation, and nutrient conversion (Blouin et al., 2013). Therefore, understanding the impact of earthworms on soil quality and their interactions with organic fertilizers is crucial for implementing sustainable soil management practices.

Previous studies have primarily focused on the replacement of chemical fertilizers with organic fertilizers to reduce chemical fertilizer inputs and obtain high crop yield (Liu et al., 2015). In this study, the aim was to assess the interactions among soil chemical properties, microbial community structure, and yield and quality of Chinese flowering cabbage under various fertilizer treatments including chemical fertilizer, earthworms, and cow manure. The study aimed to assess whether the addition of earthworms, through bioregulation, can enhance the microbial characteristics of soil that is perennially successively cropped, ultimately improving the quality and yield of Chinese flowering cabbage. In this study, our primary goals included investigating the influence of various physicochemical and biological factors on the quality and yield of Chinese flowering cabbage under equal nitrogen inputs, examining the effects of earthworms and cow manure on the soil microbial community and their underlying mechanisms, and evaluating whether earthworms and cow manure demonstrate a synergistic effect in enhancing the yield and quality of Chinese flowering cabbage under reduced chemical fertilization.

## 2. Materials and methods

### 2.1. Site description and fertilization

The experiment was performed in the No. 2 glass greenhouse of the training base of Ningxia University, Yinchuan, Ningxia (38° 5032″ N, 106° 1322″ E). The test soil was the soil under continuous cropping for 9 years at the Lijun Town, Yongning County, Yinchuan, Ningxia. The physical and chemical properties of the soil were: soil pH = 8.21, total nitrogen (TN) = 0.22 g kg^−1^, available nitrogen (AN) = 1.43 mg kg^−1^, and soil organic matter (SOM) = 6.81 g kg^−1^. The experiment was performed in potting boxes measuring 0.41 m in length, 0.27 m in width, and 0.19 m in height. Each box contained 8 kg soil with 18 cm-deep layer. The experiment involved four treatments (Table 1).

**Table 1:** Fertilizer formulation for different treatment regimens.

Treatments	Fertilizer design
CK	100% chemical fertilizer (urea, calcium superphosphate, and potassium sulfate: 3.13, 1.45, and 1.38 g/pot, respectively)
CE	30% less chemical fertilizer (urea, calcium superphosphate, and potassium sulfate: 2.19, 1.02, and 0.97 g/pot, respectively) + earthworms
CFC	30% less chemical fertilizer (urea, calcium superphosphate, and potassium sulfate: 2.19, 1.02, and 0.97 g/pot, respectively) + well-rotted cow manure
CFE	30% less chemical fertilizer (urea, calcium superphosphate, and potassium sulfate: 2.19, 1.02, and 0.97 g/pot, respectively) + well-rotted cow manure + earthworms

Each treatment was performed with six replicates. The fertilizers consisted of urea (N 46%) for N, calcium superphosphate (P_2_O_5_ 12%) for phosphorus, and potassium sulfate (K_2_O 50%) for potash. Chemical fertilizers (urea: 0.28 t/ha, calcium superphosphate: 0.13 t/ha, and potassium sulfate: 0.12 t/ha) were applied according to conventional fertilization methods. In the CFC and CFE treatments, the same nitrogen application rate was applied as in the CK treatment. The total nitrogen (TN) content of the cow manure was determined, and it was applied at the rate of 386 g/pot. The basic properties of cow manure were: water content 58%, organic carbon 163.35 g kg^−1^, and TN content 1.12 g kg^−1^. Cow manure and calcium superphosphate were used as a basal fertilizer, with 40% of urea and potassium sulfate applied as a basal fertilizer and remaining 60% as a follow-up fertilizer. Prior to the experiment, earthworms (*Eisenia foetida*) exhibiting high activity and relatively uniform size were carefully chosen. Earthworms were introduced before planting at a density of 60 g m^−2^, comprising approximately 23 red-seeded Aesop earthworms (each weighing 0.15 g). Except for the CE treatment, all treatments received the same amount of N. Chinese flowering cabbage was planted on July 10, 2022, by direct sowing at 6–7 kg/ha^−2^, Chinese flowering cabbage were interplanted after the first true leaf, harvested on August 26, 2022, and soil samples were sampled and collected.

### 2.2. Analysis of soil properties

After harvesting, plants, visible worms and insects, and stones were removed from the samples by multipoint sampling. Further, the samples were sieved through a 2 mm sieve. The soil samples were mixed, divided into multiple portions of 3–5 g each, quick-frozen in liquid nitrogen, and stored at −80°C for the determination of soil microorganisms. A portion of the soil was set aside for the determination of soil physicochemical indexes. Soil pH was determined using the potentiometric method (water:soil = 2.5:1) using a pH meter (FiveEasy Plus pH/mV, Mettler-Toledo (Schweiz) GmbH, Switzerland). Available nitrogen (AN) and TN were determined using the alkaline diffusion method and Kjeldahl digestion method, respectively. Soil available potassium (AK) and available phosphorus (AP) were extracted with CH_3_COONH_4_ solution (soil: CH_3_COONH_4_ solution = 1:10) and HCl-NH4F solution (soil: HCL-NH_4_F solution = 1:10), respectively. Soil AK was measured using a 6400A flame photometer (INESA, Shanghai, China), and soil AP was analyzed using a photometer at 660 nm. The soil organic matter (SOM) content was determined using oil bath method.

### 2.3. Soil DNA extraction and high-throughput sequencing

DNA was extracted using the E.Z.N.A. DNA Kit (Omega Bio-Tek, Norcross, GA, USA). The quality of the extracted DNA was verified using 1% agarose gel electrophoresis. DNA concentration and purity were assessed using NanoDrop 2000 spectrophotometer (Thermo Fisher Scientific). The target primer pairs were (5′-ACTCCTACG GGAGGCAGCAG-3′) and 806R (5′-GGACTACHVGGGTWTCT AAT-3′) for 16S rRNA gene in bacteria, and ITS1F (5′-CTTGG TCATTTAGAGGAAGTAA-3′) and ITS2R (5′-GCTGCGTTCTTCAT CGATGC-3′) in fungi (Zhang et al., 2020). The PCR products were separated using 2% agarose gel electrophoresis, purified with the AxyPrep DNA Gel Extraction Kit (Axygen Biosciences, Union City, CA, USA), and quantified using the QuantiFluor™-ST system (Promega, USA). Finally, bacterial and fungal amplicons were sequenced in pairs on an Illumina HiSeq sequencer. Duplicate reads were removed according to the UCHIME reference dataset, and the reads were sorted and clustered to operational taxonomic units (OTUs) using the USEARCH11-uparse algorithm with a default 97% similarity. A total of 674,096,280,450,803 bases of optimized sequences were obtained, with an average sequence length of 416 bp. All obtained raw sequence datasets have been uploaded to the NCBI Sequence Read Archive (SAR) with the accession number PRJNA1019528 (Supplementary Table S1).

### 2.4. Analysis of the quality and yield of Chinese flowering cabbage

In the harvested Chinese flowering cabbage, cabbage yield by measuring the fresh and dry weights of above- and below ground portions of cabbage. Nitrate levels were determined using the salicylic acid–sulfuric acid method. Vitamin C (VC) content was determined using 2,6-dichloroindol staining (Arya et al., 2000). The concentration of soluble sugars was determined using the anthrone sulfate method (Wang C. et al., 2020). The soluble protein content was determined using the Kormas Brilliant Blue method (Sedmak and Grossberg, 1977). To determine the nitrite levels, the vegetables samples were mixed with 0.4% p-aminobenzenesulphonic acid and 0.2% naphthylenediamine hydrochloride solution, followed by proper fixation, thorough shaking, and measurement of absorbance at 538 nm.

### 2.5. Statistical and bioinformatics analysis

Yield and quality of Chinese flowering cabbage were analyzed using one-way ANOVA in SPSS18.0 software. Estimated fungal and bacterial richness using Chao 1 indices, and the diversity index was estimated by the Shannon index. PCoA based on Bray–Curtis distance was conducted to examine the differences in microbial community composition among samples. Principal coordinate analysis (PCoA) was performed using R software (version 4.2.2). A permutation multivariate analysis of variance (PMANOVA) was performed to assess the effects of different fertilization treatments on soil microbial community structure. Species differences were analyzed using the Kruskal–Wallis rank sum test (Kruskal–Wallis *h* test). Circos mapping was conducted using Circos-0.67-67 software (Yan et al., 2020). Soil physicochemical factors and correlations between growth of Chinese flowering cabbage and soil microbial communities were assessed using mantle test in the Linket package of R software. In addition, to identify the main environmental factors affecting microbial diversity, random forest analysis was performed (Breiman, 2001), in which the relative importance of soil pH, SOM, TN, AN, AP, and AK was ranked. The random forest analysis was performed in the R statistical computing environment with the random forest package.

## 3. Results

### 3.1. Effect of different fertilizer treatments on chemical properties of soil

**Figure 1:**
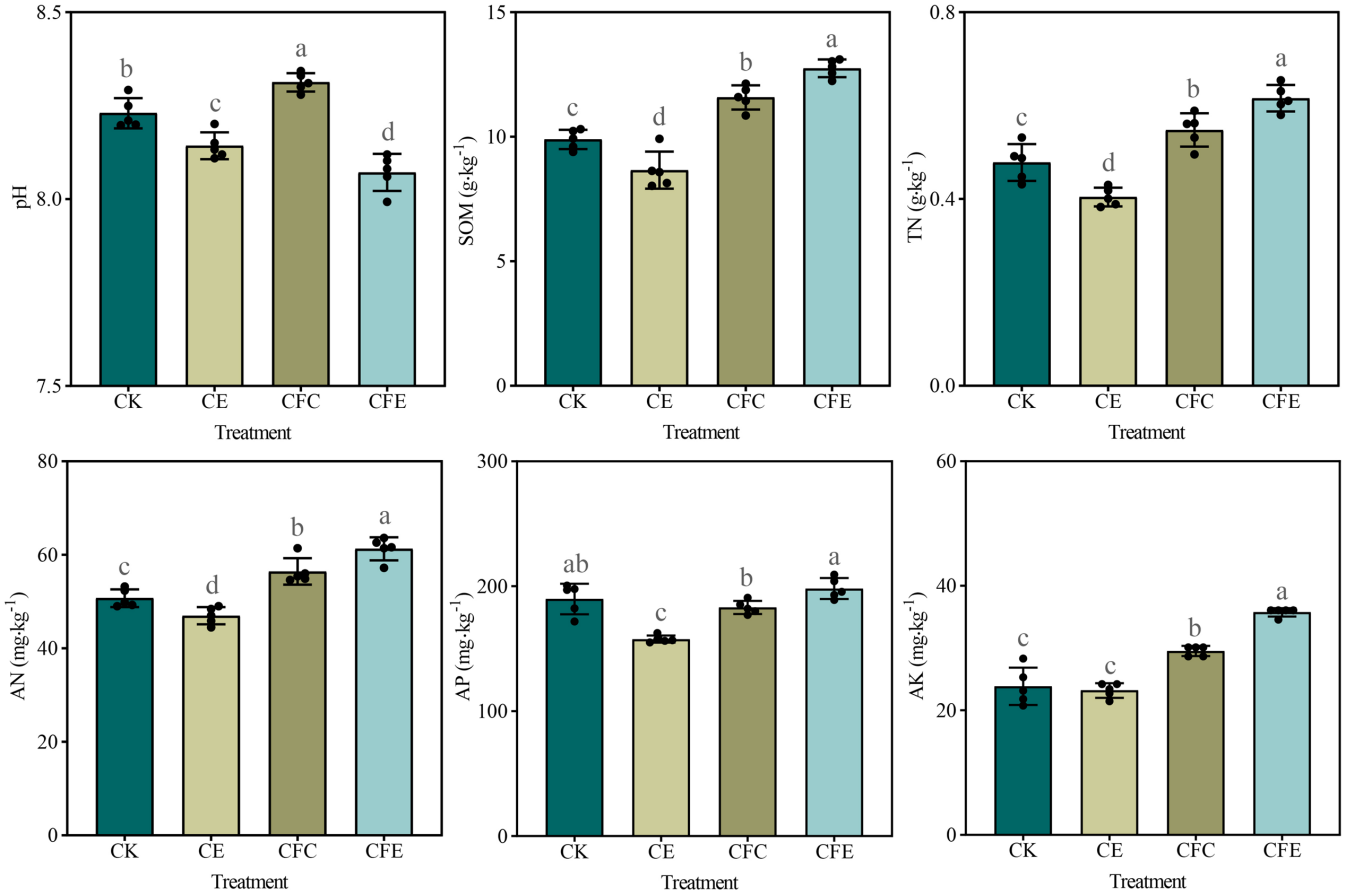
Soil physicochemical properties under different fertilizer treatments. CK: 100% chemical fertilizer; CE: 30% reduced chemical fertilizer + earthworms; CFC: 30% reduced chemical fertilizer + cow manure; CFE: 30% reduced chemical fertilizer + cow manure + earthworms. SOM: soil organic matter; TN: total nitrogen; AN: available nitrogen; AP: available phosphorus; AK: available potassium.

Inclusion of earthworms (CE) led to a reduction in soil pH, and the reduction in pH was significant when it was combined with cow manure (CFE) (*p* < 0.01). In contrast, partial replacement of fertilizer with cow manure (CFC) significantly increased soil pH (*p* < 0.05). The CE treatment decreased but both CFC and CFE treatments considerably increased SOM (Figure 1). The CFE treatment significantly changed soil AK compared with the other treatments. AP content in the CE and CFC treatments was different from that in the CFE treatment; however, AP content in the CK and CFE treatments was similar.

### 3.2. Yield and quality of Chinese flowering cabbage under different fertilizer treatments

**Figure 2:**
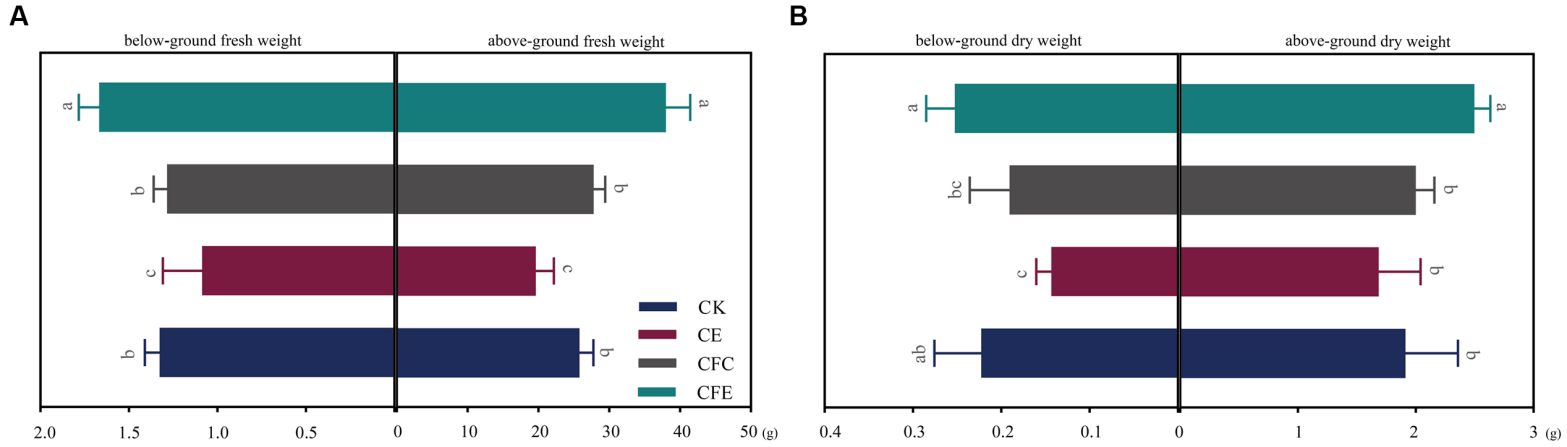
Chinese flowering cabbage yield under different treatments. Fresh weight of above- and belowground parts of Chinese flowering cabbage **(A)**. Dry weight of above- and belowground parts of Chinese flowering cabbage **(B)**. CK: 100% chemical fertilizer; CE: 30% reduced chemical fertilizer + earthworms; CFC: 30% reduced chemical fertilizer + cow manure; CFE: 30% reduced chemical fertilizer + cow manure + earthworms.

**Figure 3:**
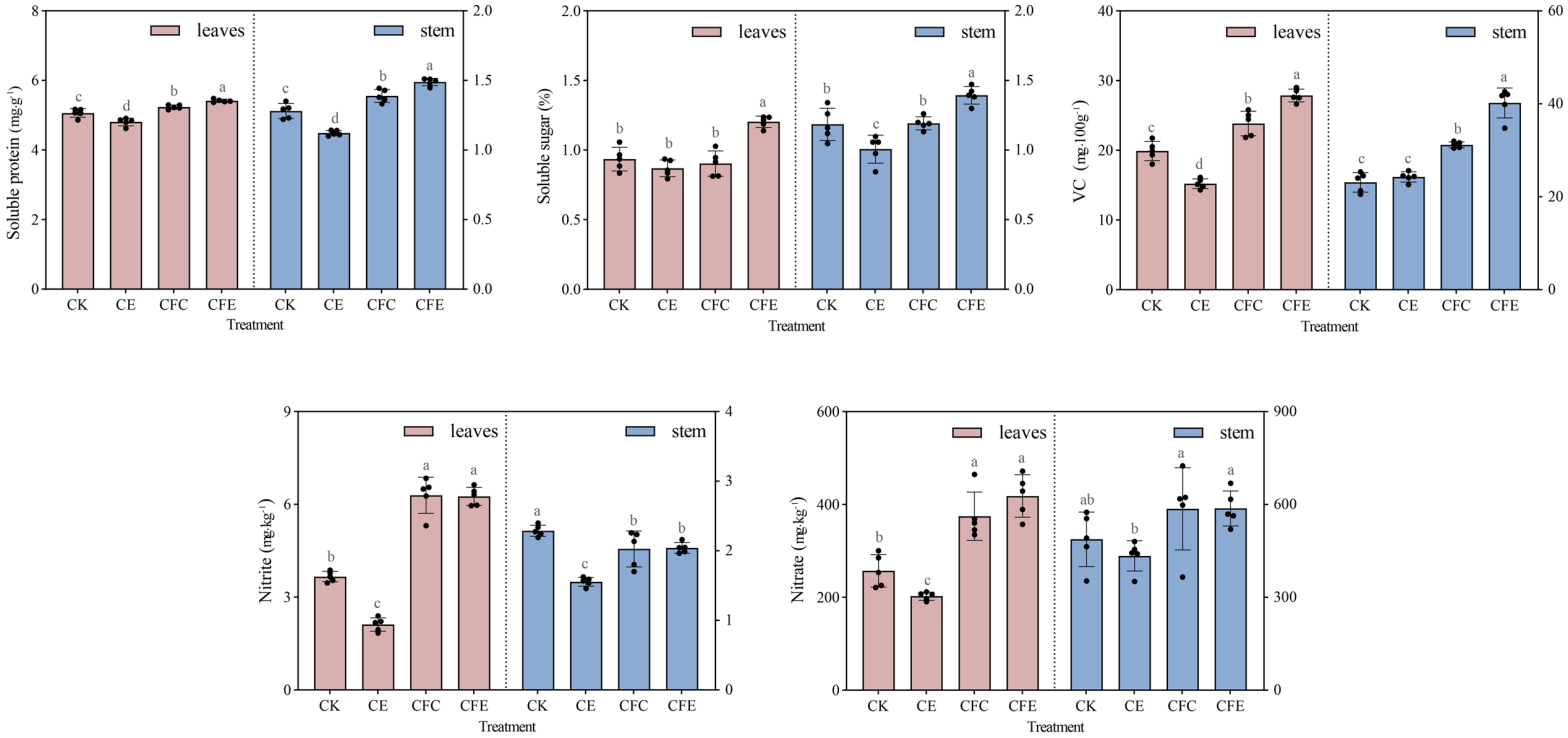
Chinese flowering cabbage quality under different treatments. CK: 100% chemical fertilizer; CE: 30% reduced chemical fertilizer + earthworms; CFC: 30% reduced chemical fertilizer + cow manure; CFE: 30% reduced chemical fertilizer + cow manure + earthworms.

The CFE treatment exhibited a significant increase in the yield of Chinese flowering cabbage, with above and below ground fresh weight of Chinese flowering cabbage exhibiting increase by 31.77 and 20.23%, respectively, compared with the CK treatment (Figure 2A). Furthermore, the CFE treatment showed significant differences in cabbage fresh weight compared to the CE and CFC treatment. In terms of aboveground dry weight, the CFE treatment resulted in 23.01% increase compared with the CK treatment, while CE and CFC treatment had no significant effect compared to CK (Figure 2B). Regarding the quality of Chinese flowering cabbage, the CFE treatment significantly enhanced soluble protein, soluble sugar, vitamin C, nitrate, and nitrite contents compared with the CK treatment (Figure 3). Similar trends were observed in the Chinese flowering cabbage stems, except for nitrite and nitrate.

### 3.3. Effect of different fertilizer treatments on the soil microbial community structure

**Figure 4:**
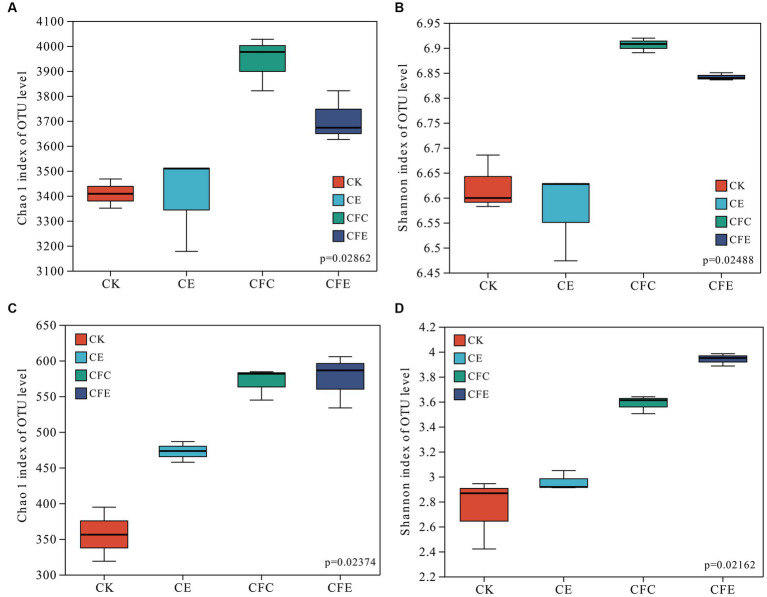
Fertilizer treatments on bacterial and fungal Chao 1 indices **(A,C)**, Shannon index **(B,D)**. CK: 100% chemical fertilizer; CE: 30% reduced chemical fertilizer + earthworms; CFC: 30% reduced chemical fertilizer + cow manure; CFE: 30% reduced chemical fertilizer + cow manure + earthworms.

The CFC and CFE treatments exhibited significant increases in both bacterial and fungal Chao 1 and Shannon indexes. However, no significant differences were observed in bacterial Chao 1 and Shannon indexes between the CE and CK treatments. The CE treatment increased fungal Chao 1 index. The bacterial Chao 1 index followed the order CFC < CFE < CE < CK, whereas the Shannon index followed the order CFC < CFE < CK < CE (Figure 4).

### 3.4. Composition of soil microbial communities

**Figure 5:**
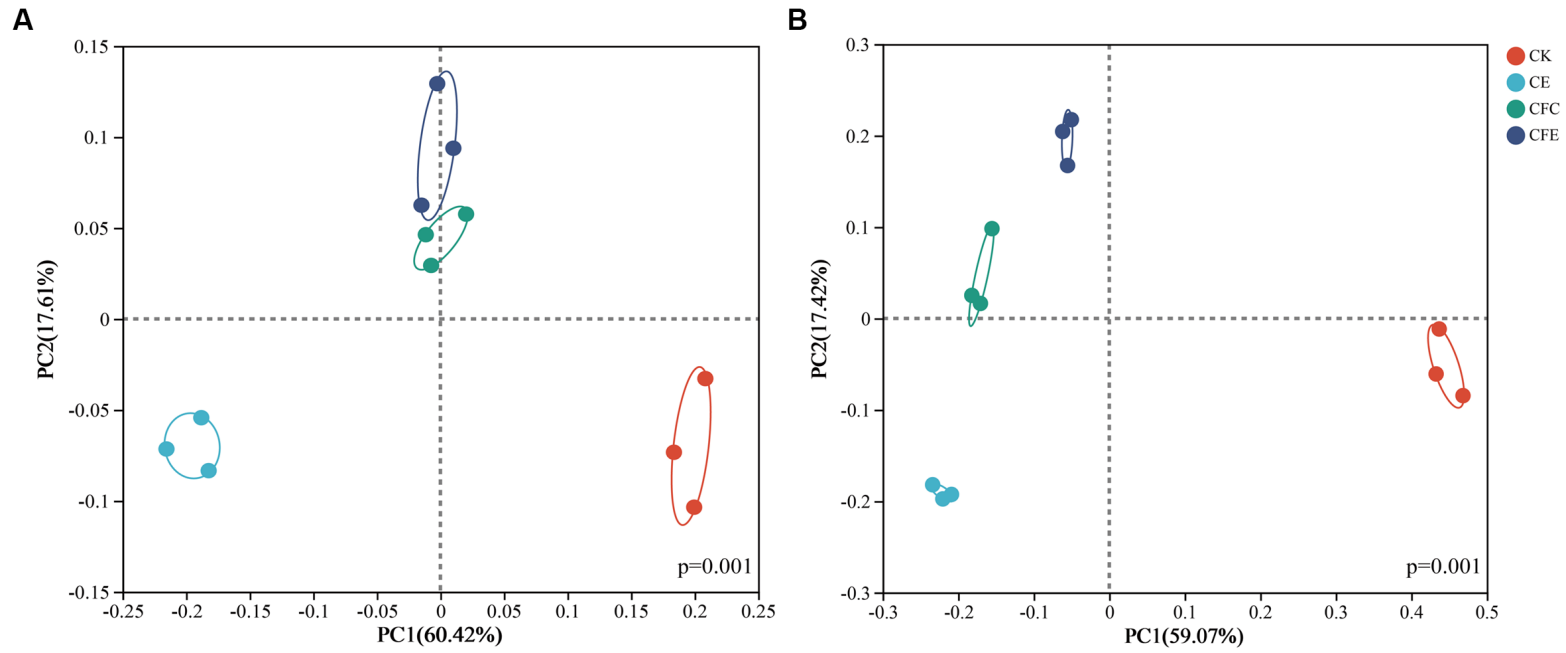
PCoA results of **(A)** bacterial (pseudo-F:8.21, *p* = 0.001, PERMANOVAR) and **(B)** fungal (pseudo-F:13.07, *p* = 0.001, PERMANOVAR) communities found with different fertilization types. CK: 100% chemical fertilizer; CE: 30% reduced chemical fertilizer + earthworms; CFC: 30% reduced chemical fertilizer + cow manure; CFE: 30% reduced chemical fertilizer + cow manure + earthworms.

Differences in soil bacterial diversity between the CFC and CFE treatments were not significant, whereas significant differences in bacterial diversity were observed between the CE, CFC, and CFE treatments compared with the CK treatment. Principal component 1 and principal component 2 accounted for 60.42 and 17.61% of the variance, respectively (Figure 5A). The treatments exhibited significant variations in fungal community composition, where principal component 1 and principal component 2 explained 59.07 and 17.42% of the variance, respectively (Figure 5B).

**Figure 6:**
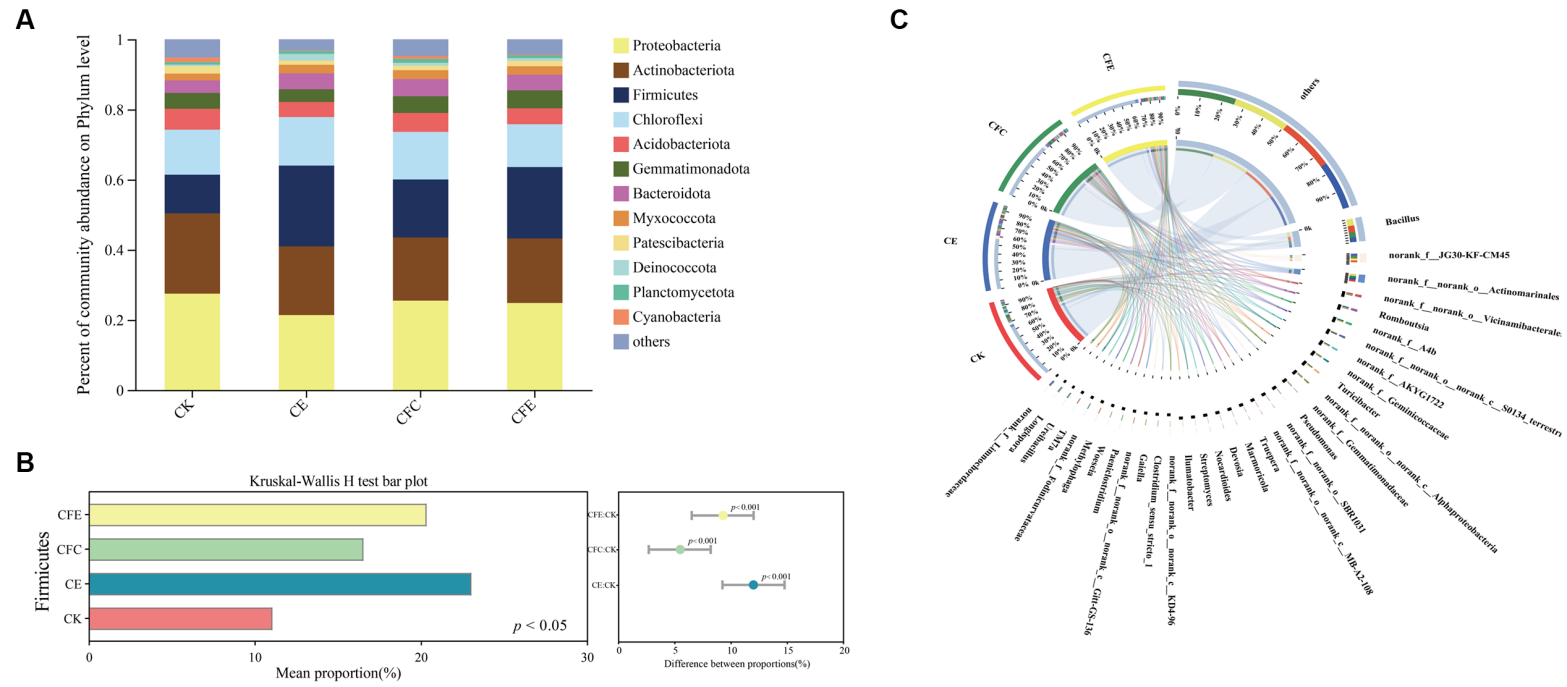
Relative abundance of bacterial taxa at the phylum level **(A)**. The relative abundances of bacteria was tested by the Kruskal–Wallis H-test **(B)**. Composition of bacterial community at the genus level **(C)**. The data were visualized by circos. CK: 100% chemical fertilizer; CE: 30% reduced chemical fertilizer + earthworms; CFC: 30% reduced chemical fertilizer + cow manure; CFE: 30% reduced chemical fertilizer + cow manure + earthworms.

The top-4 bacterial phyla identified in the four treatment groups were Proteobacteria, Actinobacteriota, Chloroflexi, and Firmicutes, representing 24.80, 19.71, 17.74, and 13.11% of the total sequences, respectively (Figure 6A). Moreover, Kruskal–Wallis *h* test demonstrated that the relative abundance of the Firmicutes clade was significantly higher in the CE, CFC, and CFE treatments compared with the CK treatment (Figure 6B; *p* < 0.05). Additionally, the relative abundance of the Firmicutes clade was higher under the CE and CFE treatments compared with the CFC treatment. Further analysis at the genus level revealed that the dominant genera were *Bacillus*, *norank_f_JG30-KF-CM45*, *norank_f_norank_o_Actinomarinales*, *norank_f_norank_o_Vicinamibacterales*, and *Romboutsia* (Figure 6C). The CFE treatment significantly increased the abundance of *Bacillus*.

**Figure 7:**
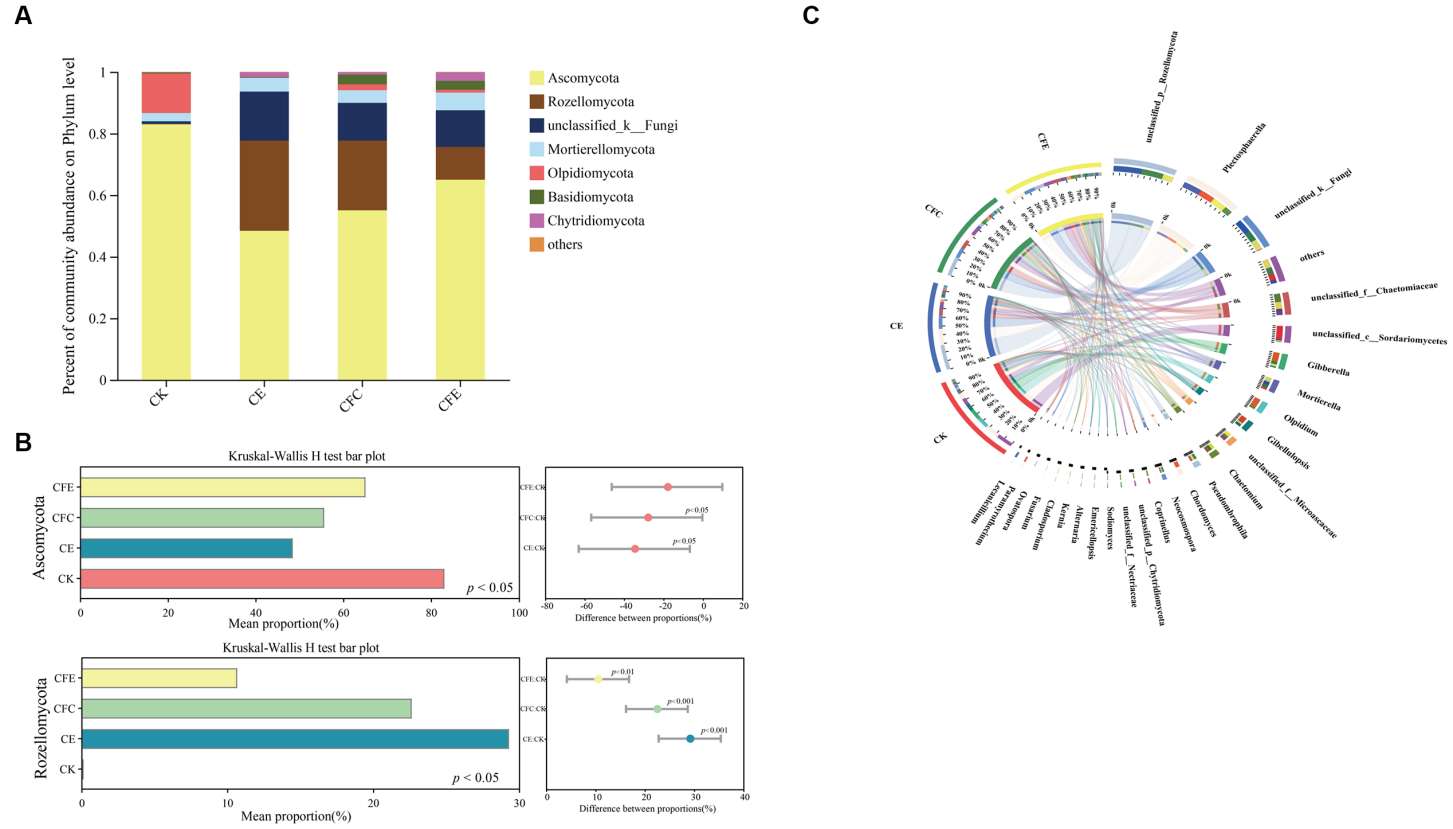
Relative abundance of fungal taxa at the phylum level **(A)**. The relative abundances of bacteria was tested by the Kruskal–Wallis H-test **(B)**. Composition of fungal community at the genus level **(C)**. The data were visualized by circos. CK: 100% chemical fertilizer; CE: 30% reduced chemical fertilizer + earthworms; CFC: 30% reduced chemical fertilizer + cow manure; CFE: 30% reduced chemical fertilizer + cow manure + earthworms.

In terms of soil fungal community composition, the dominant fungal phylum was Ascomycota, comprising 62.86% of the total sequences. It was followed by Rozellomycota, unclassified_k_Fungi, Olpidiomycota, and Mortierellomycota (Figure 7A). The relative abundance of Ascomycota was higher under the CK treatment compared with the CE, CFC, and CFE treatments. Conversely, the relative abundance of Rozellomycota and unclassified_k_Fungi was higher under the CE and CFC treatments compared with the CFE treatment. Kruskal–Wallis *h* test indicated a significantly higher relative abundance of Rozellomycota under the CE, CFC, and CFE treatments compared with the CK treatment (Figure 7B; *p* < 0.05). At the genus level, the dominant fungal genera were *unclassified_p_Rozellomycota*, *Plectosphaerella*, *unclassified_k_Fungi*, *unclassified_f_Chaetomiaceae*, *unclassified_c_Sordariomycetes*, and *Gibberella* (Figure 7C).

### 3.5. Interactions between soil physicochemistry, growth of Chinese flowering cabbage, and soil microbial communities

**Figure 8:**
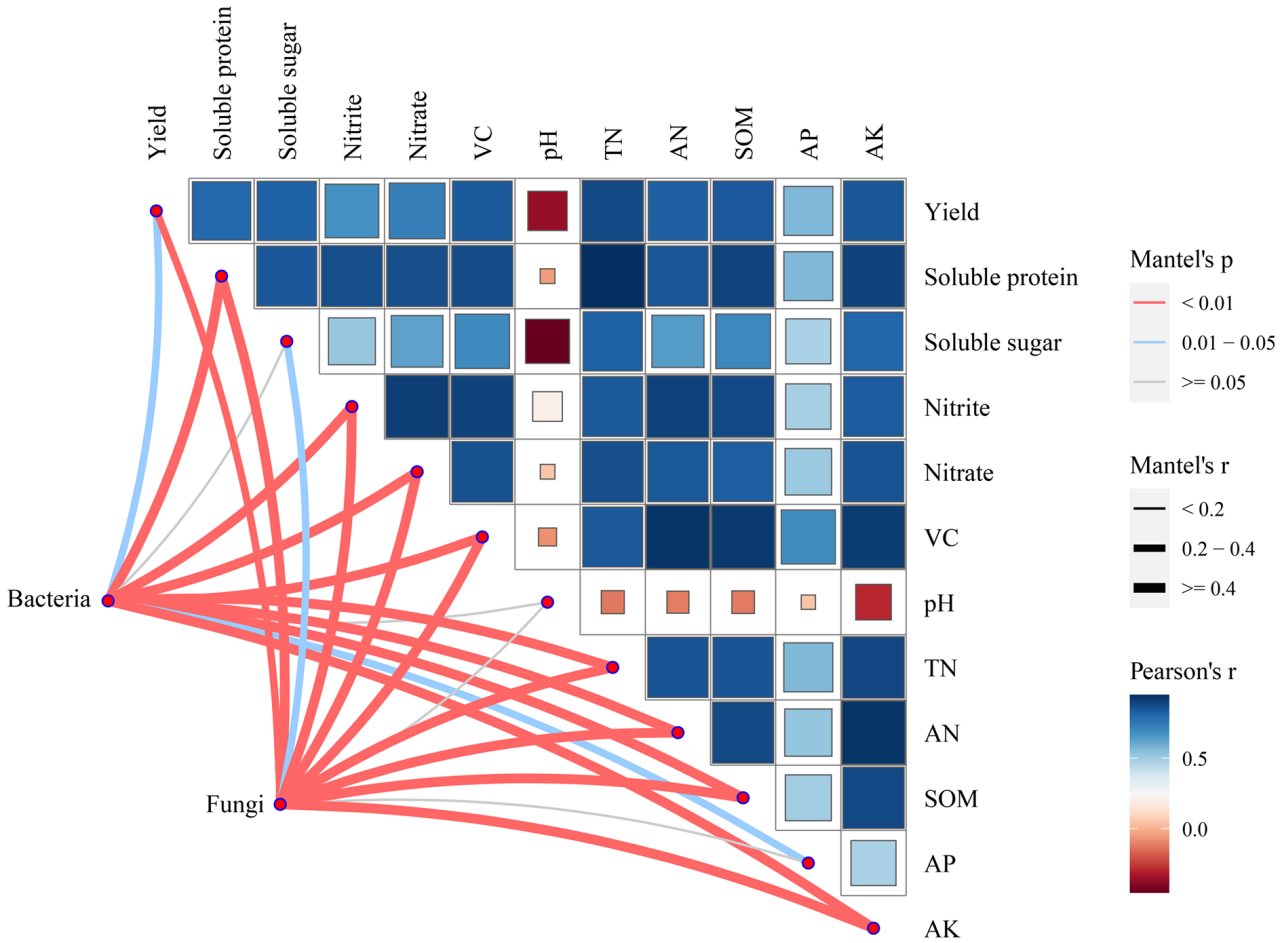
Chinese flowering cabbage growth, soil physicochemical and soil microbial interactions. CK: 100% chemical fertilizer; CE: 30% reduced chemical fertilizer + earthworms; CFC: 30% reduced chemical fertilizer + cow manure; CFE: 30% reduced chemical fertilizer + cow manure + earthworms.

**Figure 9:**
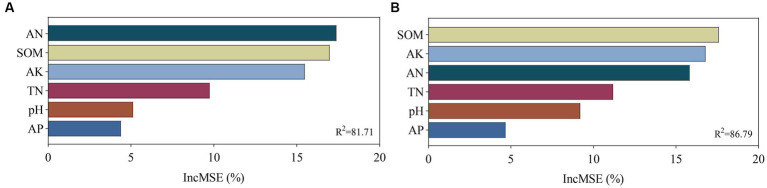
Main factors influencing microbial diversity under different treatments. This figure shows the stochastic forest importance (the percentage of increase in the mean variance error [MSE]) of soil chemical properties on the microbial diversity of bacteria **(A)** and fungi **(B)**.

The relationship among soil bacterial and fungal communities, Chinese flowering cabbage growth, and soil physicochemical properties was analyzed using mantel test. The results revealed that the yield of Chinese flowering cabbage and soluble protein, nitrite, nitrate, VC, TN, AN, SOM, AP, and AK contents were significantly correlated with bacterial communities. TN, AN, SOM, AK, yield, and all qualities of Chinese flowering cabbage were highly significantly correlated with the fungal community but pH and AP did not (Figure 8). These results were verified using random forest analysis. Random forest analysis revealed the soil bacterial community diversity was affected by AN, followed by SOM, AK, TN, pH, and AP (Figure 9A), whereas the fungal community diversity was affected by SOM, followed by AK, AN, TN, pH, and AP (Figure 9B).

## 4. Discussion

The type of fertilizer significantly influenced both soil conditions and the growth of Chinese flowering cabbage. At equal N application levels, the CFC and CFE treatments exhibited higher yield than the CK treatment. This was mainly because the reduction in the amount of chemical fertilizer was based on the nutrient uptake characteristics of Chinese flowering cabbage and soil fertility, and nutrient requirements were balanced by increasing crop nutrients (van Wesenbeeck et al., 2021). An adequate supply of TN is essential as it provides the necessary N required by plants for promoting chlorophyll synthesis and photosynthesis (Zhao et al., 2003; Liu et al., 2017). Consequently, this leads to the development of greener leaves and higher photosynthetic efficiency, which ultimately promotes plant growth and nutrient accumulation and improves the quality of Chinese flowering cabbage.

The quality of Chinese flowering cabbage was significantly improved under the combined application of earthworms and cow manure (CFE). Cow manure provided abundant nutrients and provided additional nutritional support for plant growth. This, in turn, enhanced nutrient uptake and photosynthesis (Bansal and Kapoor, 2000; Arancon et al., 2008; Guo et al., 2016). Additionally, earthworms contributed to creating a more suitable growth environment for the plant by improving soil structure, aeration, and water retention capacity (Hallam and Hodson, 2020). The results indicated that the CE treatment could not exhibit the desired improvements in the yield and quality of Chinese flowering cabbage. This can be attributed to the severe soil compaction, resulting in reduced earthworm survival, as well as lower soil porosity and water content. The application of cow manure improved soil structure by increasing organic carbon, water-soluble starch, and carbon and N contents (Kacprzak et al., 2023). In addition, the inclusion of earthworms significantly reduced soil pH. This is consistent with previous studies reporting that earthworms regulate soil pH through N excretion and calcium secretion from their glands. Additionally, the organic acids excreted by earthworms and secreted by their epidermis contribute to the reduction in soil pH (Wang G. et al., 2020). Continuous cropping of Chinese flowering cabbage increases soil pH. Therefore, inclusion of earthworms acts as a buffer, maintaining soil pH (Brautigan et al., 2014). Earthworms play a crucial role in promoting soil aggregation through their burrowing and casting activities (Lavelle et al., 2020). In this study, the CFE treatment increased SOM and soil TN, AN, and AK. The stability of soil aggregates is essential for SOM dynamics and soil fertility and reflects the influence of soil biota and soil carbon and N dynamics (Bhattacharyya et al., 2022). This influence is partly attributed to the contribution of soil biota and microorganisms to carbon and nutrient transformation at the soil aggregate scale (Vogel et al., 2022).

Soil bacterial and fungal diversity and community structure are closely related to nutrient cycling, soil quality, and productivity. Different fertilizer treatments significantly affected the alpha diversity and community structure of soil bacteria and fungi. CFC and CFE treatments increased bacterial and fungal diversity, likely due to the positive effects of earthworms and cow manure on the bacterial and fungal activities. This is consistent with previous studies. The different fertilizer treatments in this study significantly enhanced soil nutrient content, and the vast majority of Ascomycetes, Actinobacteria, and Bacteroidota (which were previously reported to be eutrophic bacteria) became the dominant species. This is consistent with a previous study reporting that the spatial distribution of the bacteria was mainly driven by nutrients (Jankowski et al., 2014). Furthermore, the abundance of Firmicutes increased after inoculation with earthworms. Previous studies have indicated that Firmicutes, as a fast-growing phylum, thrives in environments rich in carbon substrates. The continuous digging and casting activities of earthworms contribute to carbon mineralization, which explains the observed increase in the abundance of Firmicutes after the inclusion of earthworms (Singh et al., 2016; He et al., 2020).

The Chao 1 and Shannon indexes were lower, and the abundance of *Bacillus* was higher in the CFE treatment than in the CFC treatment (Liu et al., 2021). The higher abundance of the *Bacillus* in the CFE treatment than in the other treatments can be attributed to the fact that this genus is mostly aerobic or partially anaerobic photosynthetic bacteria, and earthworms can form loose and porous vermicompost due to their own feeding and movement of the earthworm haptosphere (Przemieniecki et al., 2021). *Norank_f_JG30-KF-CM45* was negatively correlated with TOC. This suggested that inoculation with earthworms can increase the decomposition rate of organic matter and improve the quality of organic fertilizer (Jiang et al., 2020). *Norank_f_norank_o_Actinomarinales* had the lowest abundance in the CK treatment. Actinomyces spp. promoted organic matter conversion, and the abundance of Actinomyces spp. increased after inoculation with earthworms and cow manure, which participated in soil nutrient cycling and improved soil fertility (Janvier et al., 2007). This confirmed that earthworms can enhance the beneficial effects of organic fertilizers on the abundance, activity, and community structure of soil microorganisms (Huang et al., 2014).

Among fungi, Ascomycota was the most abundant mycorrhizal fungal phylum in the CK treatment, with a decrease in abundance after inoculation with earthworms or addition of organic fertilizer. This is consistent with previous studies suggesting that the relative abundance of soil fungal phyla varies depending on cropping patterns (Wang et al., 2019). The significant abundance of Ascomycota may reflect the unique distribution pattern of fungi in agricultural soils, particularly where plant diversity is particularly low. The CFE treatments increased the abundance of Basidiomycota (Figure 7). A competitive relationship may exist between the ascomycetes and the Stramenopiles resulting in a decrease in the abundance of Ascomycota (Ye et al., 2020). Compared with the CK treatment, all other treatments significantly increased the abundance of Rozellomycota and *unclassified_p_Rozellomycota*. Interestingly, Rozellomycota is frequently detected in animal gut (Li et al., 2020). This suggested that earthworms or organic fertilizers may have a more pronounced positive effect on Rozellomycota compared with chemical fertilizers. At the fungal genus level, the CFE and CFC treatments reduced the abundance of *Plectosphaerella* and *Gibberella*, taxa that are known to be the major pathogens responsible for root and stem rot in many plant species (Farh et al., 2018). Inoculation with earthworms significantly increased the abundance of *unclassified_k_Fungi* and *unclassified_f_Chaetomiaceae*, which have cellulose-degrading capacity, and altered the community structure of soil fungi, which play a role in soil ecological cycle system (Song et al., 2020). Previous studies have associated *Gibberella* with severe decay of leaves, peduncles, and flowers in potted hyacinths and with cob rot disease (Erysipelas cob rot) in maize (Tomioka et al., 2008; Tian et al., 2021). The CFE treatment reduced the abundance of *Gibberella*. Various studies have reported that alterations in soil microbial communities are closely related to the inhibition of soilborne pathogenic fungi (Wang et al., 2017). Therefore, inoculation of earthworms and cow manure can inhibit the enrichment of these pathogenic fungi, thereby reducing the incidence of root rot and other diseases.

Soil microbial diversity and communities can significantly influence soil quality and the sustainability of soil ecosystems. Previous studies have reported that soil pH is an important factor in microbial community structure. However, in this study, this effect did not appear to be significant. Soil pH was not significantly correlated with bacterial and fungal communities and was negatively correlated with the yield and quality of Chinese flowering cabbage. This may be attributed to the fact that soil pH is alkaline because of the perennial continuous cropping of Chinese flowering cabbage; however, the optimum growth environment for bacteria is lower neutral, which resulted in no significant correlation between pH and bacterial community (Fernández-Calviño et al., 2011). Fungi are only slightly affected by soil pH as they have a strong ability to adapt to acidity (Nevarez et al., 2009; Rousk et al., 2010). Random forest analysis indicated that AN, SOM, AK, and TN were the main factors affecting bacterial and fungal diversities and indicated a significant positive correlation with the quality and yield of Chinese flowering cabbage. These physicochemical factors could partially explain the nutrient cycling and utilization by microorganisms in the ecosystem (Zhao et al., 2019). Numerous studies have indicated that more than 50% of the N required by crops is obtained from soil, whereas the remainder is derived from in-season fertilizer applications. In soil, soil organic N constitutes approximately 90% of the TN content (Xu et al., 2016). This is consistent with previous studies consistently demonstrating that the application of organic fertilizer significantly enhances soil organic N and SOM content, thereby promoting plant growth (Zhou et al., 2013). Abundant nutrients such as organic N and organic potassium in SOM provide a sustained and balanced supply of nutrients to crops (Han et al., 2021). These nutrients are gradually released into inorganic forms through microbial decomposition and mineralization, effectively meeting the nutrient requirements for crop growth and quality development (Tahat et al., 2020). Overall, different fertilization treatments altered the microbial diversity and community in the soil by regulating the soil properties, and the combined effect of earthworms and cow manure promoted the growth of Chinese flowering cabbage and optimized the soil structure.

## 5. Conclusion

This study revealed that the synergistic effect of earthworms and cow manure under reduced application of chemical fertilizer decreased soil pH; increased soil organic matter, total nitrogen, available nitrogen, and available potassium; and effectively improved soil chemical properties. This study indicated that the synergistic effect of earthworms and cow manure promoted the growth of Chinese flowering cabbage and increased microbial diversity and altered community structure. Earthworms and cow manure promoted the abundance of *Bacillus* and decreased the abundance of *Plectosphaerella* and *Gibberella*. This study provided a scientific basis for the establishment of environmentally friendly fertilization techniques to effectively promote sustainable agricultural development.

## Data availability statement

The original contributions presented in the study are publicly available. This data can be found here: NCBI - PRJNA1019528.

## Author contributions

FG: Writing – original draft, Writing – review & editing. LY: Methodology, Resources, Writing – review & editing. XM: Writing – review & editing. LX: Investigation, Writing – review & editing. ZS: Investigation, Writing – review & editing. YL: Investigation, Writing – review & editing.
